# Abnormal phenotype of Nrf2 is associated with poor prognosis through hypoxic/VEGF-A-Rap1b/VEGFR2 pathway in gastric cancer

**DOI:** 10.18632/aging.204013

**Published:** 2022-04-13

**Authors:** Ya Yang, Xin Wang, Jia Zhang, Hao Gu, Song Zhang, Hao Sun, Junqi Liu, Ruitai Fan

**Affiliations:** 1 Department of Radiotherapy, The First Affiliated Hospital of Zhengzhou University, Zhengzhou 450000, Henan, China; 2 Department of Thoracic Surgery, The First Affiliated Hospital of Xi'an Jiaotong University, Xi’an 710061, Shaanxi, China

**Keywords:** gastric cancer, Nrf2, Rap1b, hypoxic, prognosis

## Abstract

Metastasis is the major cause of death in gastric cancer patients and altered expression of Nrf2 is associated with cancer development. This study assessed Nrf2 and HO-1 expression and hypoxia-induced Nrf2 expression in the promotion of metastatic potential of gastric cancer cells, the relationship of Rap1b and Nrf2 was also discussed. Nrf2 and HO-1 expression were significantly associated with clinicopathological characteristic and were independent prognostic predictors in gastric cancer patients. Hypoxia up-regulated the expression of Nrf2, HO-1 and HIF-1α, whereas knockdown of Nrf2 inhibited cell invasion capacity and reduced the expression of Nrf2, HO-1 and HIF-1α. Patients in the Rap1b (+) Nrf2 (+) group had worst overall survival compared with those from other groups. Knockdown of Rap1b and Nrf2 significantly inhibited cell invasion capacity in the common group compared with the other groups. Hypoxia or VEGF-A facilitated the nuclear translocation of Nrf2 through Rap1b or VEGFR2. Hypoxia or VEGF-A did not induce the phosphorylation of P-Erk1/2 and P-Akt after knockdown of Rap1b or VEGFR2. Hypoxia promoted the gastric cancer malignant behavior through the upregulation of Rap1b and Nrf2. Hypoxia/VEGF-A-Rap1b/VEGFR2 facilitated the nuclear translocation of Nrf2. Targeting Rap1b and Nrf2 may be a novel therapeutic strategy for gastric cancer.

## INTRODUCTION

Gastric cancer (GC) is one of the most important malignancies globally; it is the fifth most commonly diagnosed and the third major cause of cancer-related mortality [1]. In the previous decades, although the GC incidence rate was experiencing a steady decline globally, the median survival was less than 12 months for the advanced stage [2, 3]. Given that the early symptoms of GC are not obvious, GC is often diagnosed at an advanced stage and has a poor prognosis. Recent data indicates that the prevalence of GC is particularly high in Eastern Asian countries such as China, Japan, and Korea [4, 5]. Studies have documented that GC is a highly invasive heterogeneous malignant tumor. Gene mutations, epigenetic changes, and abnormal molecular signaling cascades participate in the development, diffusion, and metastasis of GC. Identification of the molecular profile of GC can provide milestone progress in achieving effective treatment and enhancing the quality of life, as well as reducing the mortality rates of GC patients.

Nrf2 is a member of the NF-E2 family of transcription factors with a basic leucine zipper. Kelch-like ECH-associated protein 1 (Keap1) acts as a cysteine-rich suppressor protein and docks to Nrf2. In normal conditions, Keap1 inhibits Nrf2 activity; however, under oxidative stress, Nrf2 dissociates from Keap1, promoting the activation of Nrf2. Nrf2 positively regulates anti-inflammatory, antioxidant, detoxification and drug resistance effects to protect normal cells from oxidative stress and exogenous stimuli [6]. In addition, Nrf2 participates in various cell activities, such as cancer cell proliferation and invasion, apoptosis, chemoresistance, cell cycle repair, tissue regeneration, and immune regulation [7]. Nrf2 is overexpressed in a variety of tumor cells and has been correlated with the occurrence and progression of many tumors, including advanced lung cancer, squamous cell carcinoma of the head and neck, liver cancer, esophageal squamous cell cancer, colon cancer, pancreatic cancer, gastric cancer, and gallbladder cancer [8, 9]. Nrf2 may be a remarkable signature for the prognosis of individuals with GC and play an indispensable role in the onset and progress of GC.

The association between Nrf2 and GC has been reported in several studies, but the significance of Nrf2 in GC metastases and invasion is not well elucidated. Hence, we explored the relationship between Nrf2 and GC, as well as possible mechanisms and signaling pathways. Hypoxia is known to play an important role in the TME (tumor microenvironment) to impact tumor cell survival, as well as malignant behaviors [10, 11]. Therefore, we explored the relationship between hypoxia and Nrf2, as well as related mechanisms in the invasion of GC. Previous investigations found that Rap1b and hypoxia-inducible factor-1 alpha (HIF-1) protein expression contribute to the GC malignant behavior along with poor prognosis [12]. Nevertheless, the function of Rap1b and its crosstalk with Nrf2 in GC remains unclear.

## RESULTS

### The clinicopathological data of GC patients is related to the differential expression of the proteins Nrf2 and HO-1

**Figure 1:**
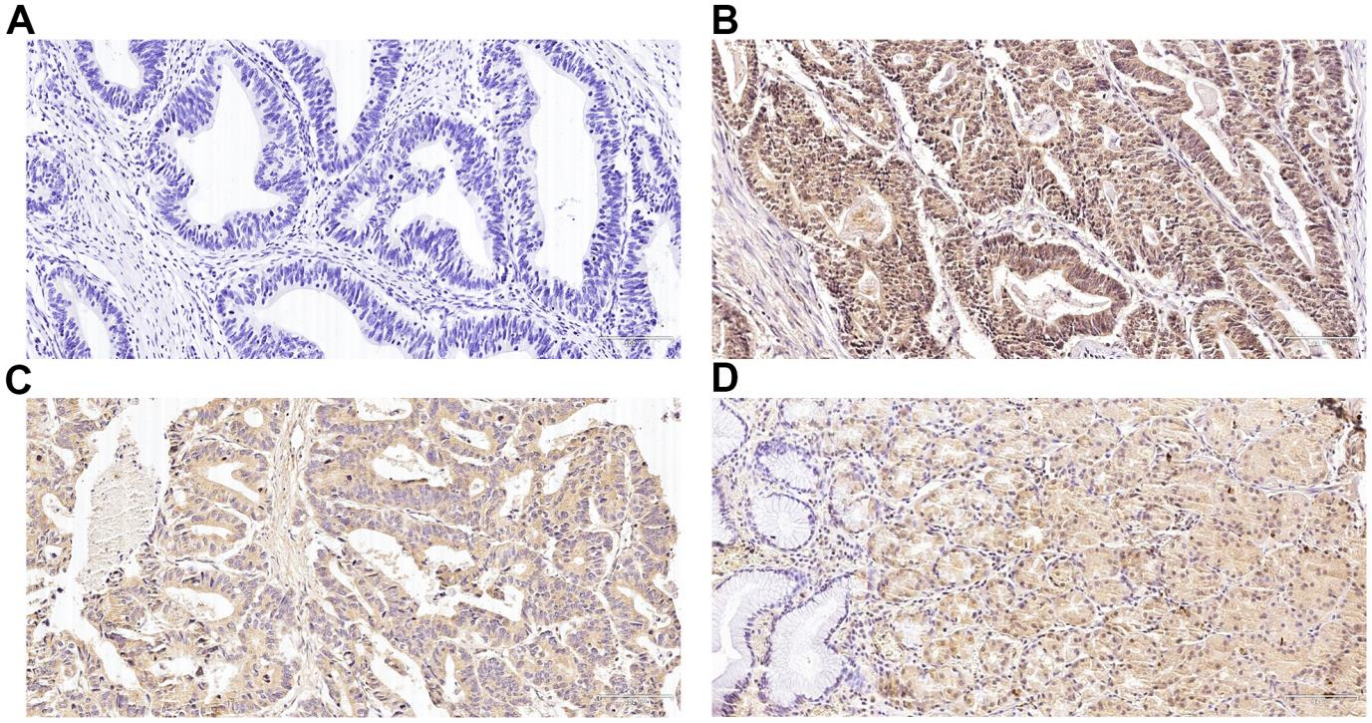
**Immunohistochemical expression of Nrf2 and heme oxygenase-1 (HO-1) in gastric cancer (GC) and para-carcinoma tissues.** (**A**) Slides without primary antibody served as the negative control in GC tissues. (**B**) Typical immunohistologic features with Nrf2 expression in GC tissues. The Nrf2 staining localized predominantly in the nucleus. (**C**) Typical immunohistologic features with HO-1 expression in GC tissues. (**D**) Typical immunohistologic features with Nrf2 expression in para-carcinoma tissues. Magnifications, ×200.

In GC tissues, Nrf2 was mainly localized in the nucleus (Figure 1B) and concomitant cytoplasmic staining was partly observed. In para-cancer normal tissues, Nrf2 was primarily located in the cytoplasm and HO-1 was predominantly expressed in the cytoplasm (Figure 1C). In cancer tissues, the positive expression rate of Nrf2 was 73.59% (131/178), and the positive expression rate of HO-1 was 69.66% (124/178). Nrf2 along with HO-1 proteins expression was remarkably different between GC and neighboring non-malignant tissues (Nrf2, P<0.001; HO-1, P<0.001) (Table 1). Expression of Nrf2 protein was remarkably associated with the depth of infiltration (P<0.001), regional lymph nodes (P<0.001), distant metastasis (P<0.001), Borrmann type (P<0.001) along with TNM staging (P =0.014) but not with gender, age, tumor differentiation or tumor diameter (Table 2). Similar results were observed for HO-1. In addition, Nrf2 expression was positively related with the expression of HO-1 (r = 0.605, P<0.001) and HIF-1α (r =0.446, P<0.001) (previous studies [12]; Table 3).

**Table 1:** Differences in Nrf2 and HO-1 between the cancer tissues and para-carcinoma tissues.

**Group**	**Nrf2**		***P***	**HO-1**		***P***
	**+**	**-**		**+**	**-**	
cancer	131	47		124	54	
para-carcinoma	30	148	< 0.001	65	113	< 0.001
total	161	195		189	167	

**Table 2:** Correlations between the clinicopathologic variables with Nrf2 and HO-1.

**Variables**	**N**	**Nrf2**		***P***	**HO-1**		***P***
		**High**	**Low**		**High**	**Low**	
Gender							
Male	125	82	43		73	52	
Female	53	31	22	0.397	30	23	0.824
Age							
≤ 60	94	58	36		53	41	
> 60	84	55	29	0.602	50	34	0.672
TNM							
I	21	9	12		9	12	
II	34	20	14		15	19	
III	83	52	31		51	32	
IV	40	32	8	<0.001	28	12	<0.001
Depth of invasion							
T1	16	4	12		6	10	
T2	18	12	6		7	11	
T3	67	48	19		43	24	
T4	77	49	28	<0.001	47	30	<0.001
Nodal involvement							
N0	49	26	23		22	27	
N1	36	18	18		18	18	
N2	35	22	13		22	13	
N3	58	47	11	<0.001	41	17	<0.001
Metastasis							
M0	138	81	57		73	65	
M1	40	32	8	0.014	30	10	0.013
Borrmann type							
I	22	12	10		10	12	
II	55	31	24		31	24	
III	62	44	18		39	23	
IV	39	26	13	<0.001	23	16	<0.001
Differentiation							
High	12	7	5		5	7	
Moderate	57	34	23		31	26	
Poor	109	72	37	0.667	67	42	0.340
Tumor diameter							
<3cm	44	22	22		20	24	
3cm-5cm	54	38	16		33	21	
>5cm	80	53	27	0.090	50	30	0.156

**Table 3:** Association between Nrf2 with HO-1 and HIF-1α.

	**Nrf2**		**r**	***P***
	**High**	**Low**		
HO-1				
high	91	22		
low	12	53	0.605	< 0.001
HIF-1α				
high	80	33		
low	16	49	0.446	< 0.001

### Association of Nrf2 and HO-1expression with patient survival

**Figure 2:**
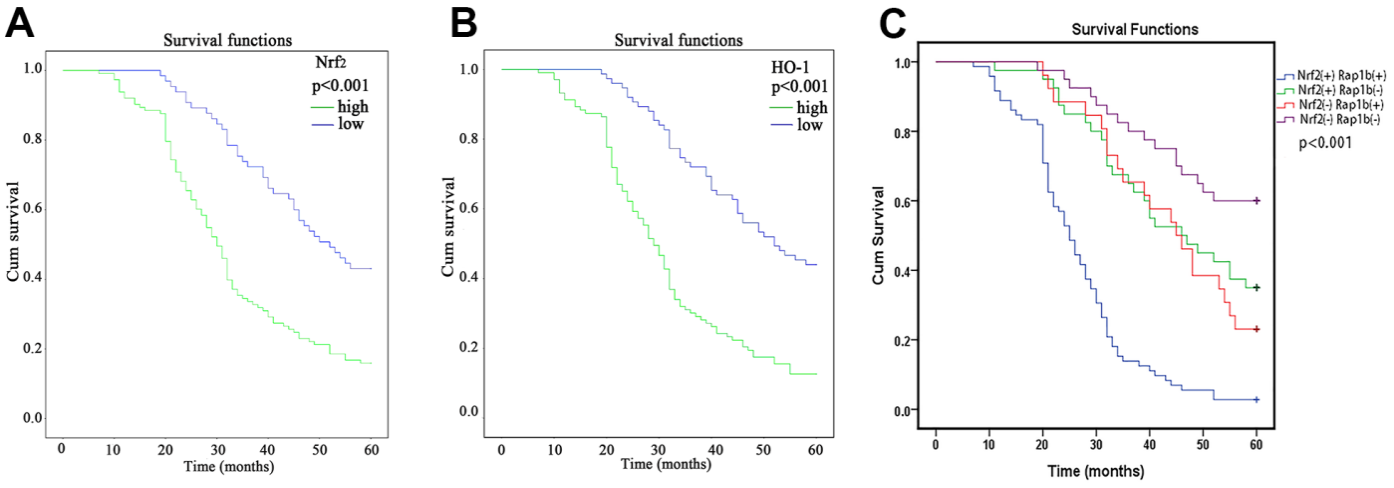
**Kaplan-Meier survival analysis of Nrf2, HO-1 and Rap1b expression levels.** (**A**) Differences in the cumulative OS among patients with Nrf2 protein expression. (**B**) Differences in the cumulative OS among patients with HO-1 protein expression. (**C**) Differences in cumulative OS among patients with Nrf2 and Rap1b protein expression. The P-value was obtained using the log-rank test of the difference. (**P<0.001).

The results of the Kaplan-Meier curve and log-rank test exhibited a significant difference in overall survival (OS) between patients with high Nrf2 expression and those with low Nrf2 expression (Figure 2A and Table 4), 30 months (95% CI, 27.52-32.48) vs. 52 months (95% CI, 42.97-61.02, P<0.001), respectively. Similar results were observed for HO-1 expression (P<0.001, Figure 2B and Table 4). In addition, the multivariate Cox data illustrated that overexpression of Nrf2 (P = 0.035, RR = 1.583, 95% CI 1.033-2.426) and HO-1 (P<0.001, RR = 2.156, 95% CI 1.428-3.257) were independent predictive factors for the survival of individuals with GC (Table 5).

**Table 4:** Univariate analysis for overall survival.

**Variables**	**N**	**Overall survival**		**P**
		**Median±SE**	**95%CI**	
Nrf2				
High	113	30.00 ± 1.26	27.52–32.48	
Low	65	52.00 ± 4.60	42.97–61.02	< 0.001
HO-1				
High	103	29.00 ± 1.56	25.94–32.06	
Low	75	52.00 ± 5.77	40.68–63.31	<0.001
Gender				
Male	125	34.00±2.97	28.16-39.83	
female	53	38.00±3.63	30.86-45.13	0.973
Age				
≤60	94	40.00±3.87	32.40-47.59	
>60	84	32.00±1.13	29.77-34.22	0.223
TNM				
I	21	49.00±3.21	42.69-66.71	
II	34	45.00±5.83	33.57-56.42	
III	83	34.00±2.27	29.53-38.46	
IV	40	29.00±1.57	25.91-32.08	<0.001
Depth of invasion				
T1	16	46.00±8.66	29.01-62.98	
T2	18	52.00±15.91	20.81-83.18	
T3	67	35.00±4.60	25.98-44.01	
T4	77	31.00±1.45	28.14-33.86	0.005
Nodal involvement				
N0	49	55.00±9.79	35.79-74.20	
N1	36	43.00±7.80	27.22-58.78	
N2	35	36.00±3.54	29.04-42.95	
N3	58	29.00±2.53	24.03-33.96	<0.001
Metastasis				
M0	138	40.00±3.91	32.32-47.67	
M1	40	29.00±1.57	25.91-32.08	<0.001
Borrmann type				
I	22	39.00±7.62	24.06-53.93	
II	55	40.00±3.69	32.76-47.23	
III	62	34.00±3.50	27.14-40.85	
IV	39	30.00±1.77	27.51-34.48	0.502
Differentiation				
high	12	40.00±6.06	28.11-51.88	
moderate	57	39.00±6.11	26.97-51.02	
poor	109	32.00±1.74	28.59-35.41	0.107
Tumor diameter				
<3cm	44	46.00±5.20	35.79-56.20	
3cm-5cm	54	34.00±1.83	30.40-37.51	
>5cm	80	32.00±0.98	30.06-33.93	0.019

**Table 5:** Multivariate Cox proportional hazards analysis for overall survival.

**Variables**	**Overall survival**		***P***
	**RR**	**95%CI**	
TNM	1.615	1.309–1.994	< 0.001
HO-1	2.156	1.428-3.257	< 0.001
Nrf2	1.583	1.033–2.426	0.035

### Nrf2 and HO-1 are upregulated in GC cells under hypoxic conditions

**Figure 3:**
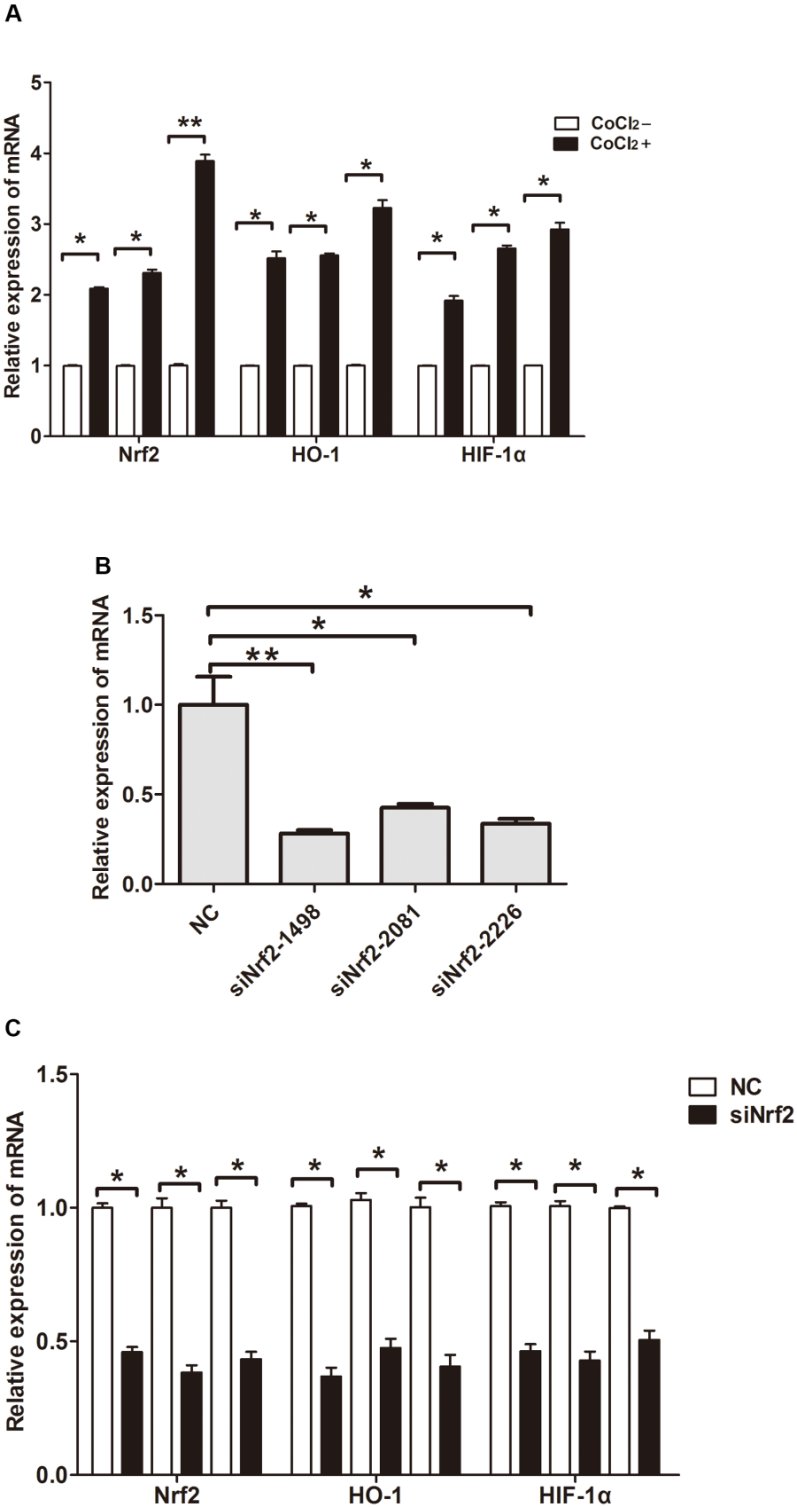
**Relative expression of mRNA.** (**A**) Expression of Nrf2, HO-1 and HIF-1α in GC cells cultured under a hypoxic condition. (**B**) Expression of Nrf2 in GC cells after they were transfected with negative control siRNA or Nrf2 siRNAs (siNrf2-1498, siNrf2-2081 and siNrf2-2226). (**C**) Expression of Nrf2, HO-1 and HIF-1α in GC cells after they were transfected with siNrf2-1498 and cultured under a hypoxic condition. (*P<0.05, **P<0.001).

**Figure 4:**
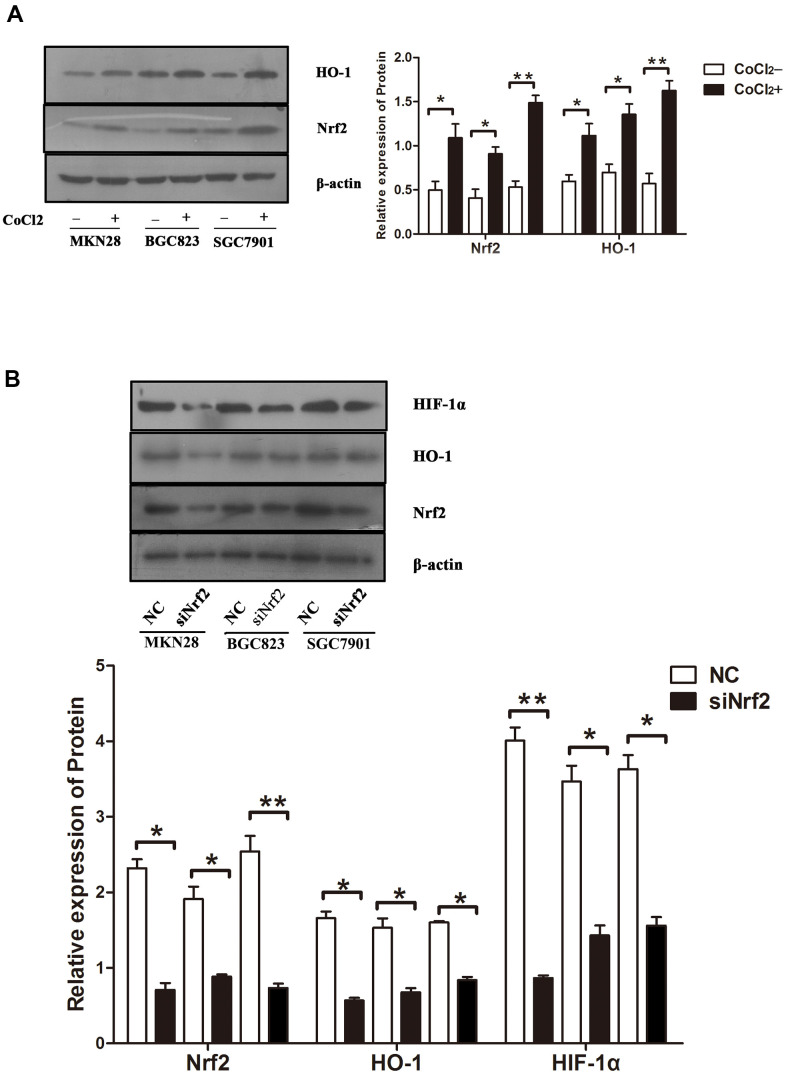
Protein expression under different conditions (**A**) Expression of Nrf2 and HO-1 in GC cells cultured under a hypoxic condition. The graphs show the quantified data of western blots. (**B**) Expression of Nrf2, HO-1 and HIF-1α in GC cells after they were transfected with siRNAs and cultured under a hypoxic condition. The graphs show the quantified data of western blot. (*P<0.05, **P<0.001).

Previous studies confirmed that hypoxia could induce GC cell infiltration and increase HIF-1α expression at the protein level [12]. To verify whether hypoxia increases Nrf2 along with HO-1 expression and induces the invasion of GC cell, MKN28, BGC823 as well as SGC7901 cells were treated with 150 or 200 μM CoCl2 for 24 h. Our findings indicated an increase in Nrf2 and HO-1 mRNA and protein expression (Table 6 and Figures 3A, 4A, P<0.05). HIF-1α was up-regulated at the mRNA level (Table 6 and Figure 3A, P<0.05). Therefore, hypoxia can increase the expression of Nrf2, HO-1, and HIF-1α in GC cells.

**Table 6:** The primer sequences.

**Primer**	**Sequence**
Nrf2	5’ TCAGCGACGGAAAGAGTA TGA 3’, 5’ CCACTGGTTTCTGACTGGATG T 3’
HO-1	5’ CATCACCAGCTTAAAGCCTT 3’ 5’ CAGGCAGAGAATGCTGAGTTC 3’
HIF-α,	5’ GAACGTCGAAAAGAAAAGTCTCG 3’ 5’ CCTTATCAAGATGCGAACTCACA 3’
VEGFR2,	5’ TCCGTTTCCTGCAGCAGTCTCCGCA 3’ 5’ AGAGAGCCAGCCCTCTGACGTCCAT 3’
VEGF-A	5’ CACCGCCTCGGCTTGTCACAT 3’ 5’ CTGCTGTCTTGGGTGCATCTG 3’
GAPDH	5’ CTGGGGCTCATTTG 3’ 5’ CATCCACAGTCTTC 3’

### Silencing the expression of Nrf2 inhibits the invasion of GC cells under hypoxic conditions

**Figure 5:**
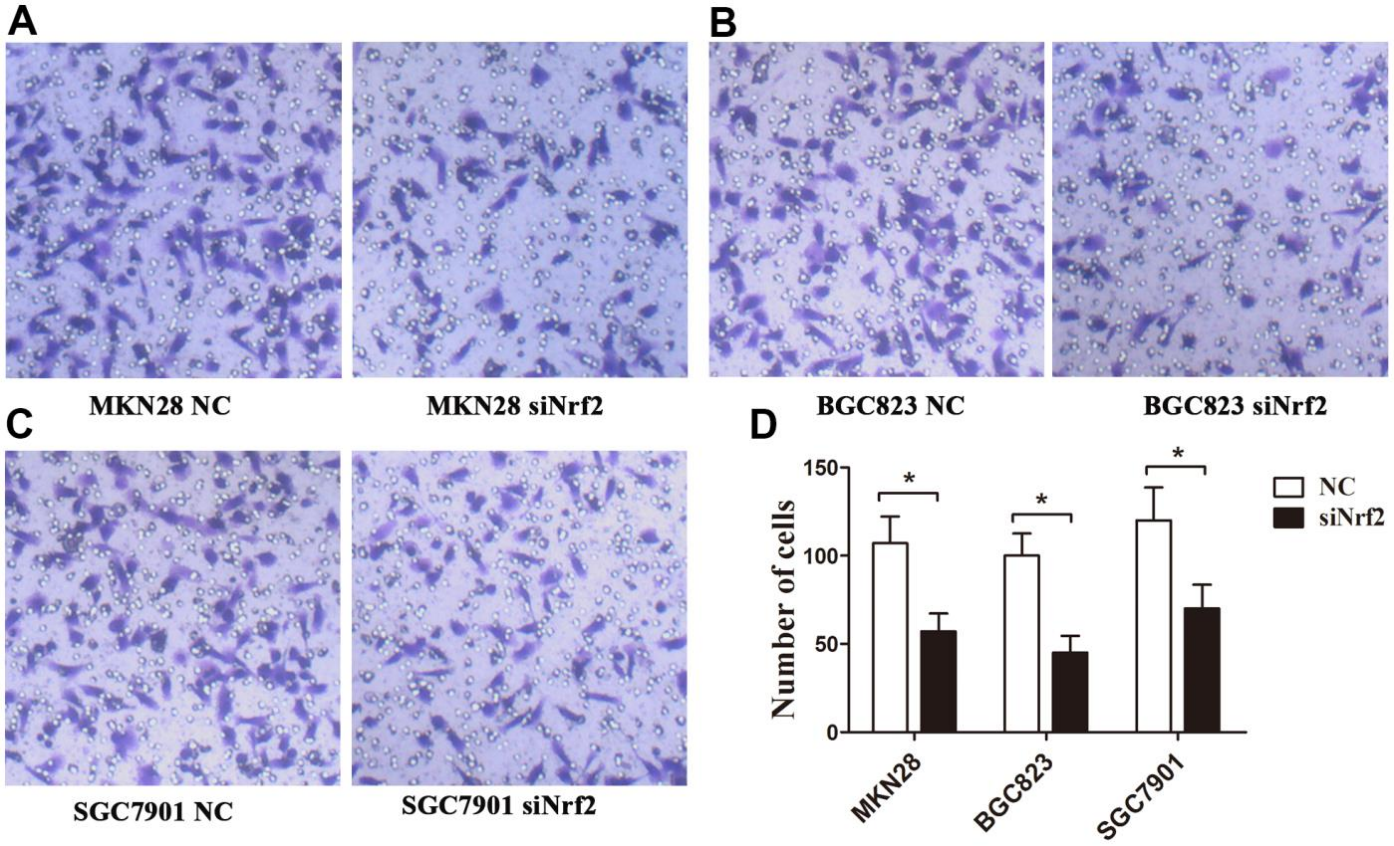
**Effects of Nrf2 knockdown on the regulation of the GC invasion capacity under a hypoxic condition.** The invasion capacity was suppressed in GC cells transfected with siNrf2-1498 under hypoxic conditions. (**A**) MKN28 cells; (**B**) BGC823 cells; (**C**) SGC7901 cells; (**D**) The graph shows the quantified data of the assay. (**P<0.001).

To clarify the function of Nrf2 in GC cells, we found that siNrf2-1498 was the most effective sequence (Table 7 and Figure 3B). Therefore, we used a negative control and siNrf2-1498 to transfect GC cells, processed the cells with 150 or 200 μM CoCl_2_ for 24 h, and then conducted the transwell invasion experiment. Under hypoxic conditions, the invasive potential of MKN28, BGC823, as well as SGC7901 cell transfects of siNrf2-1498 was reduced remarkably (P<0.05, Figure 5). Silencing of Nrf2 expression repressed HO-1 and HIF-1α expression in hypoxic conditions (P<0.05, Figures 3C, 4B). Therefore, Nrf2 facilitates the infiltration potential of GC cells in a hypoxic environment.

**Table 7:** The small interference RNA sequences of human Nrf2 and VEGFR2.

**Gene**	**Sequence**
Nrf2-homo-2081	5’ GCUGCUCAGAAUUGCAGAATT 3’ 5’ UUCUGCAAUUCUGAGCAGCTT 3’
Nrf2-homo-1498	5’ GCCCAUUGAUGUUUCUGAUTT 3’ 5’ AUCAGAAACAUCAAUGGGCTT 3’
Nrf2-homo-222	5’ GCACCUUAUAUCUCGAAGUTT 3’ 5’ ACUUCGAGAUAUAAGGUGCTT 3’
Negative control siRNA	5’ UUCUCCGAACGUGUCACGUTT 3’ 5’ ACGUGACACGUUCGGAGAATT 3’
VEGFR2-homo-366	5’ GGGUUUGCCUAGUGUUUCUTT 3’ 5’ AGAAACACUAGGCAAACCCTT 3’
VEGFR2-homo-633	5’ CUCGGUCAUUUAUGUCUAUTT 3’ 5’ AUAGACAUAAAUGACCGAGTT 3’
VEGFR2-homo-990	5’ GUCUCAUGGAAUUGAACUATT 3’ 5’ UAGUUCAAUUCCAUGAGACTT 3’

### Relationship between Rap1b and Nrf2

**Figure 6:**
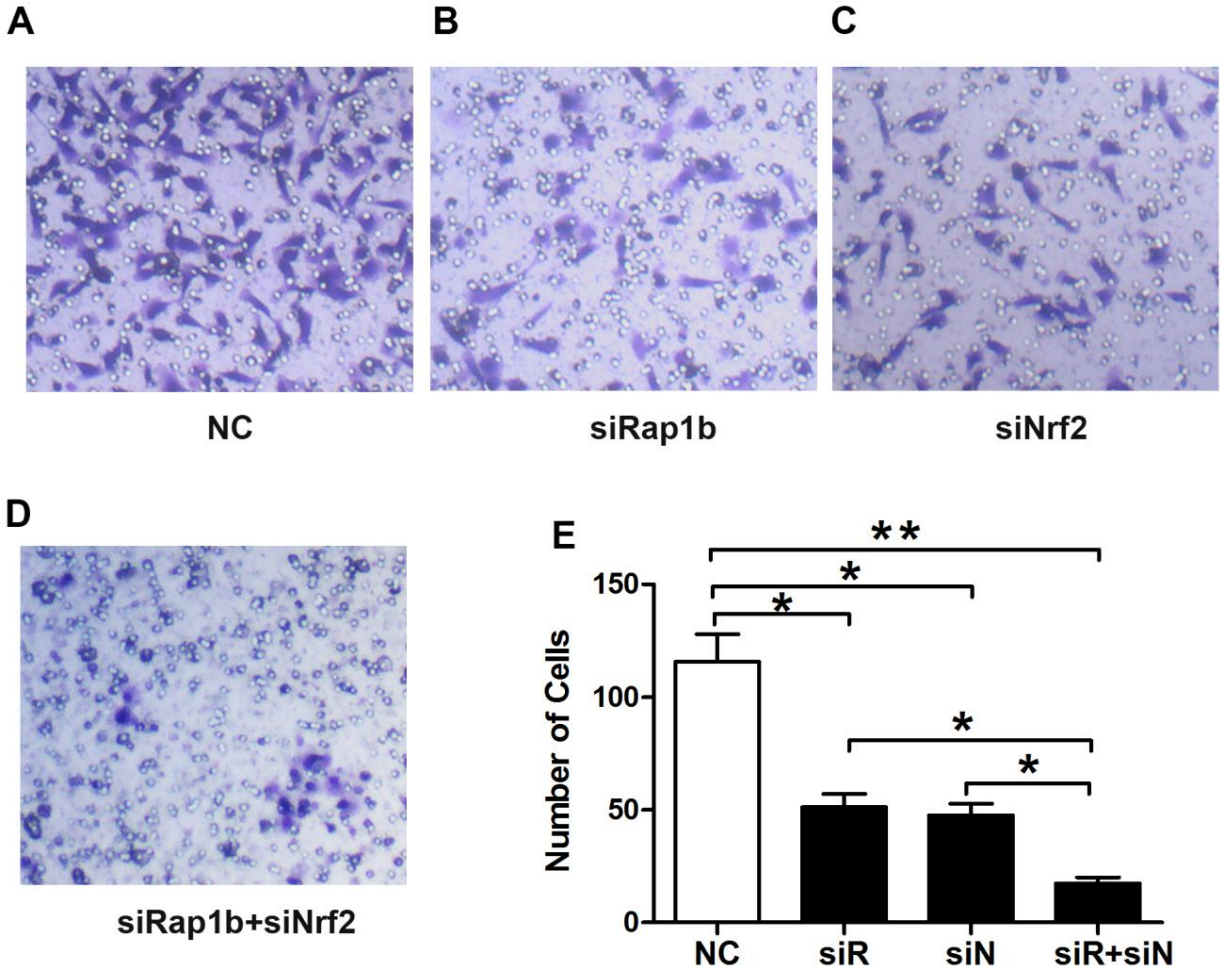
**Effects of Nrf2 or (and) Rap1b knockdown on the regulation of the GC invasion capacity under a hypoxic condition.** The invasion capacity was suppressed in GC cells transfected with siNrf2RNA-1498 or (and) siRap1bRNA-501 under hypoxic conditions. (**A**) Negative control; (**B**) siRap1bRNA; (**C**) siNrf2RNA; (**D**) siRap1bRNA and siNrf2RNA; (**E**) The graph shows the quantified data of the assay. (*P<0.05, **P<0.001).

Rap1b and Nrf2 can promote the invasion and metastasis of GC. Whether the two have a synergistic effect on the progression of GC remains unclear. To confirm the correlation between Rap1b and Nrf2, Spearman’s analysis showed that the two were positively correlated (r = 0.581, P<0.001, Table 8). Univariate survival assessment exhibited that in contrast with patients from other groups, patients in the Nrf2 (+) Rap1b (+) group had the worst survival (OS 26.58 ± 1.30, 95% CI 24.02-29.14) (Table 9 and Figure 2C). Rap1b and Nrf2 can be used as joint markers to assess the prognosis of GC patients. Furthermore, the silencing of Rap1b and Nrf2 remarkably repressed the tumor cell infiltration capacity in the common group in contrast with the other groups, especially in the negative control group (Figure 6).

**Table 8:** Association between Nrf2 and Rap1b.

	**Rap1b**		**r**	***P***
	**High**	**Low**		
Nrf2				
high	87	26		
low	11	54	0.581	< 0.001

**Table 9:** Univariate analysis for overall survival.

**Variables**	**N**	**Overall survival**		***P***
		**Median±SE**	**95%CI**	
Nrf2(+)Rap1b(+)	72	26.58 ±1.30	24.02–29.14	< 0.001
Nrf2(+)Rap1b(-)	40	44.37 ±2.36	39.74–49.00	
Nrf2(-)Rap1b(+)	26	43.96 ±2.59	38.88–49.04	
Nrf2(-)Rap1b(-)	40	50.90 ±2.01	46.94–54.85	

### Nuclear translocation of Nrf2

**Figure 7:**
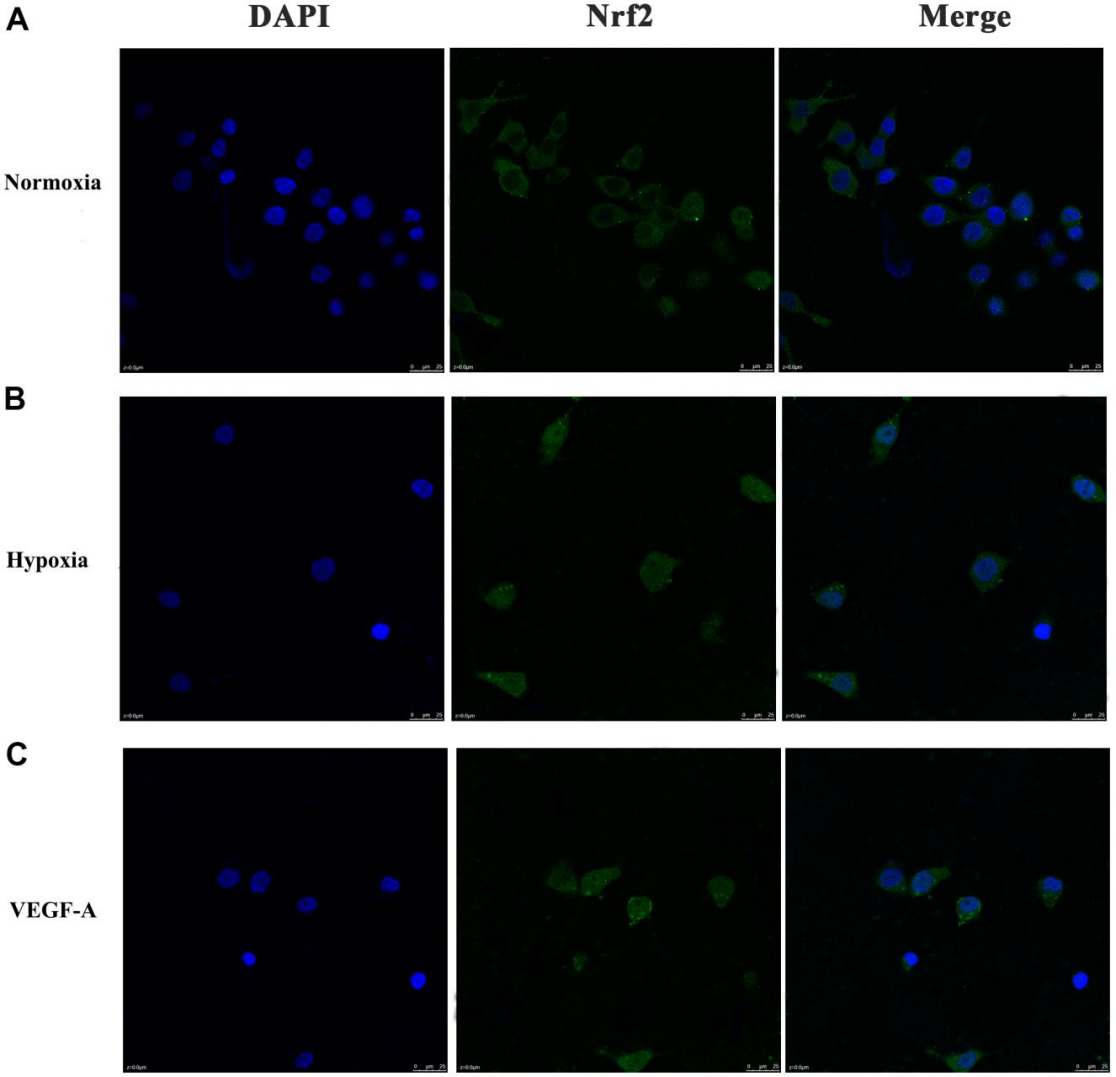
**Subcellular localization of Nrf2 in GC cells.** (**A**) Normoxia (**B**) Hypoxia (**C**) VEGF-A (×1000).

**Figure 8:**
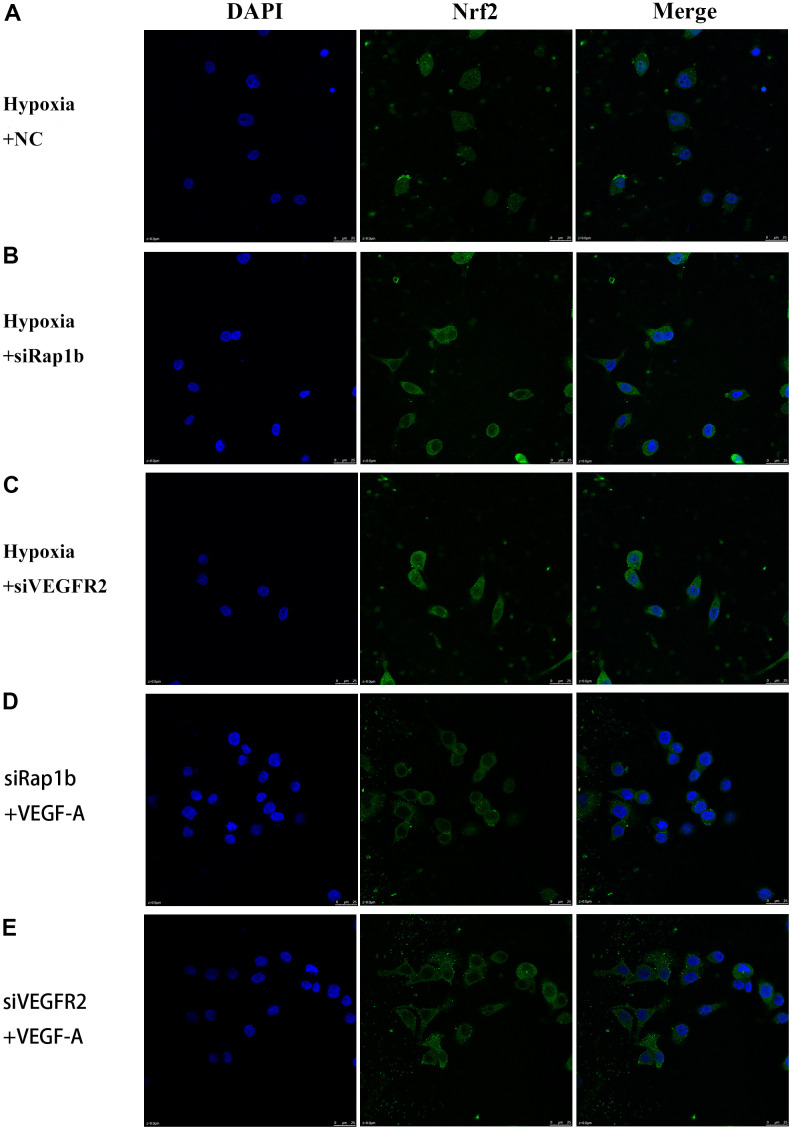
**Subcellular localization of Nrf2 in GC cells.** (**A**) Hypoxia and negative control. (**B**) Hypoxia and siRap1b. (**C**) Hypoxia and siVEGFR2. (**D**) siRap1b and VEGF-A. (**E**) siVEGFR2 and VEGF-A (×400).

**Figure 9:**
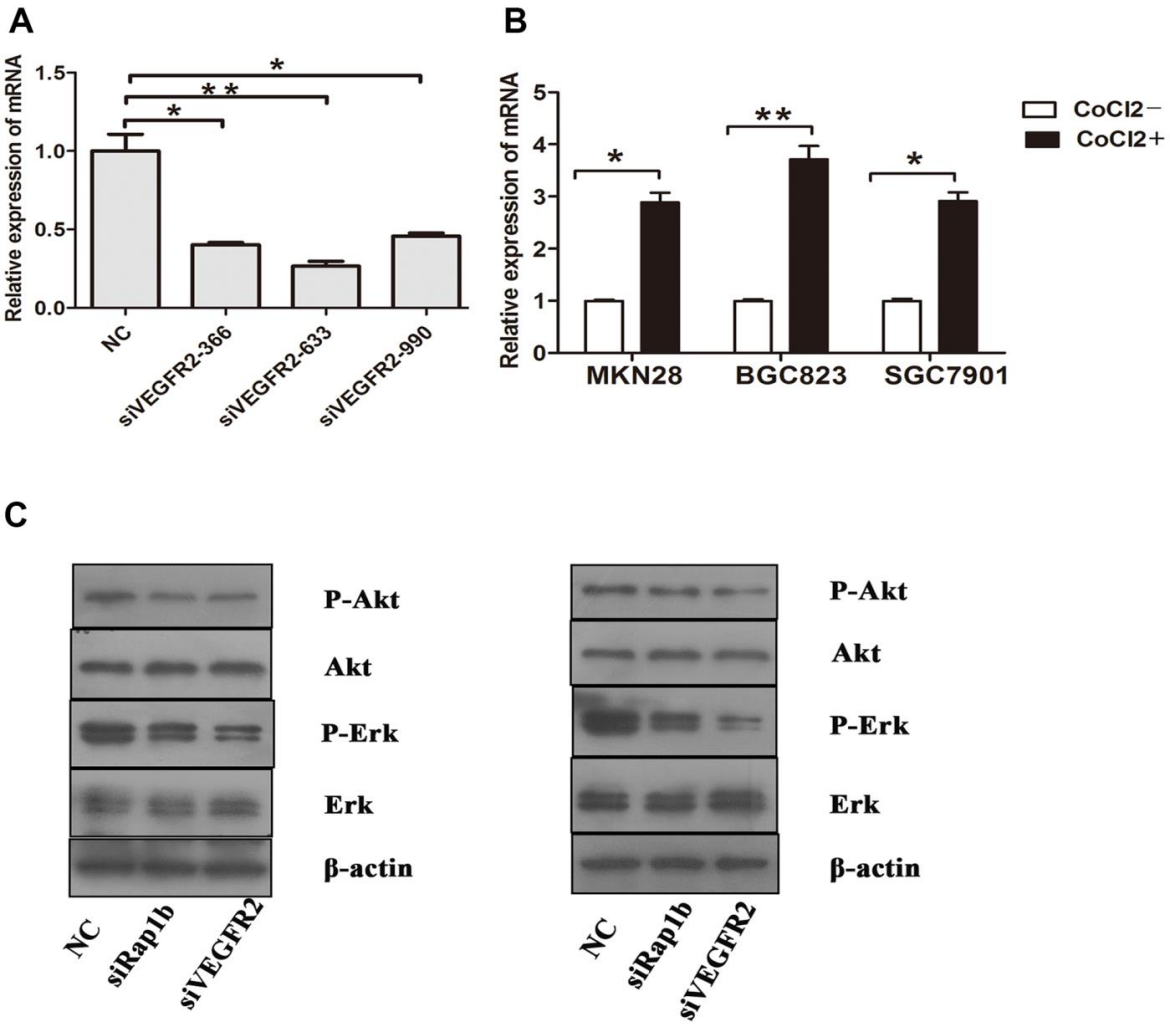
Relative expression of mRNA and the phosphorylation of protein (**A**) Expression of VEGFR2 in GC cells after they were transfected with negative control siRNA or VEGFR2 siRNAs (siVEGFR2-366, siVEGFR2-633 and siVEGFR2-990). (**B**) Expression of VEGF-A in GC cells cultured under a hypoxic condition. (**C**) Expression of Erk, P-Erk, Akt and P-Akt in GC cells after transfected with siRap1b or siVEGFR2 (left) Hypoxia (right) VEGF-A. (*P<0.05, **P<0.001).

**Figure 10:**
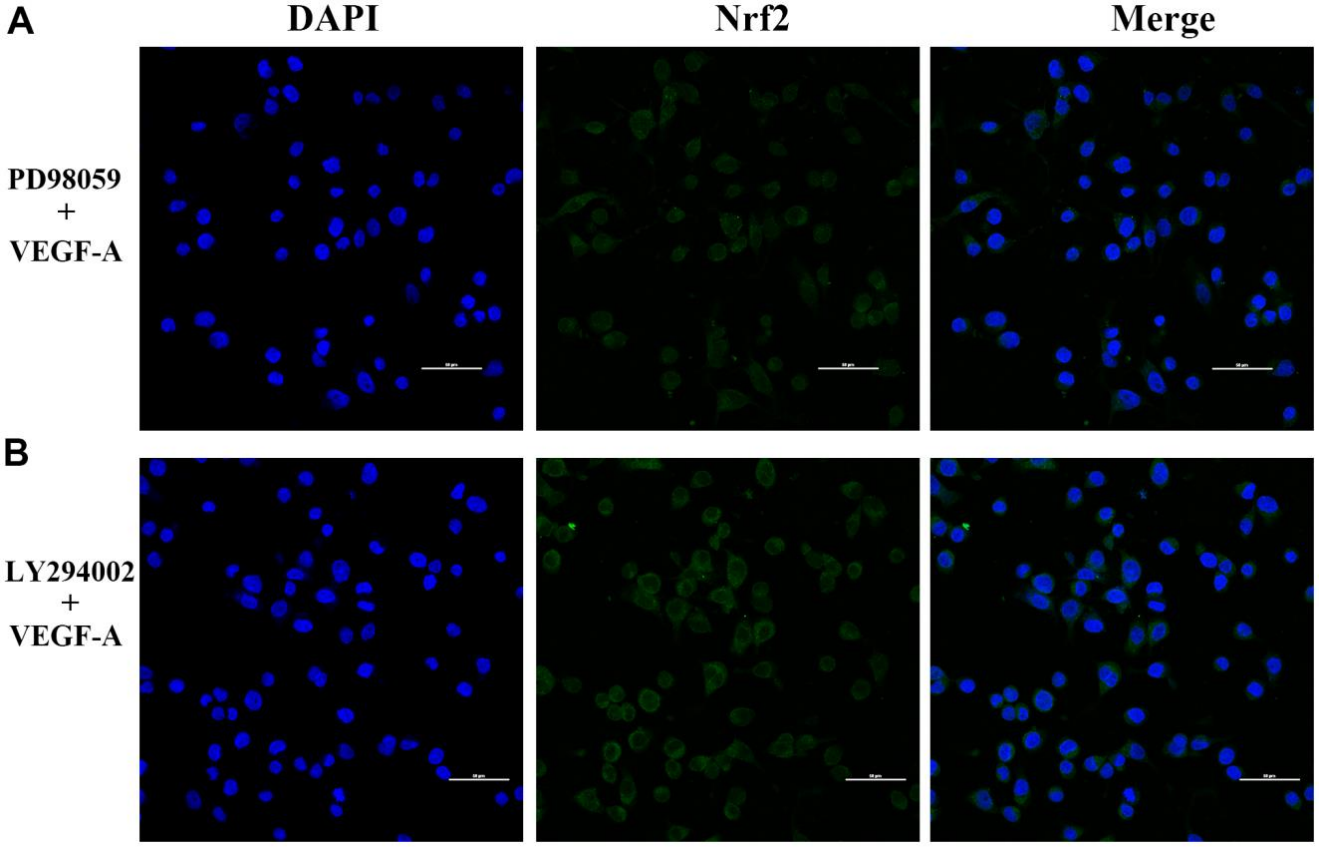
**Subcellular localization of Nrf2 in GC cells.** (**A**) PD98059 and VEGF-A (**B**) LY294002 and VEGF-A (×400).

**Figure 11:**
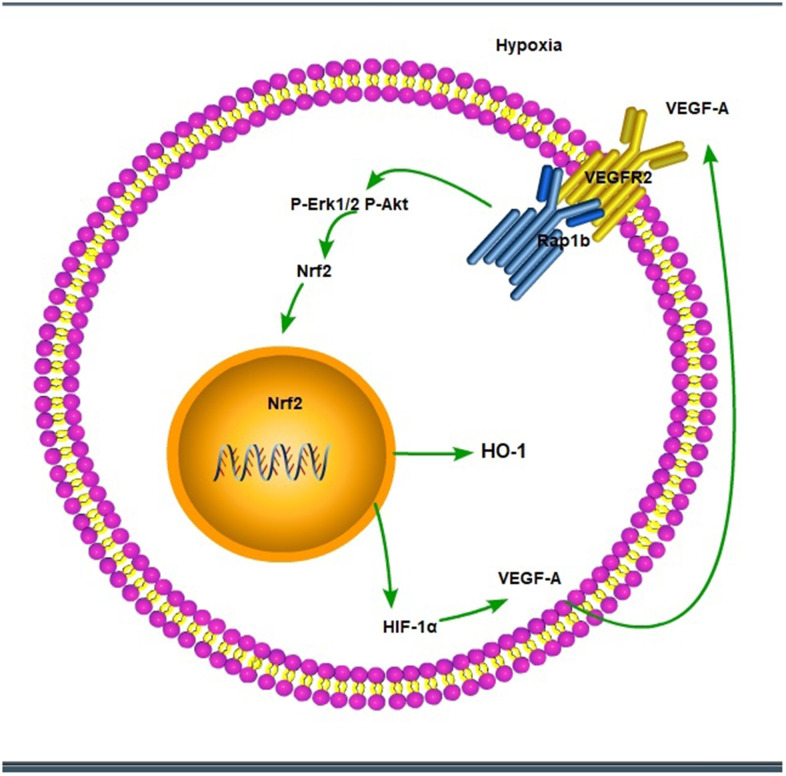
The scheme of hypoxic/VEGF-A-Rap1b/VEGFR2 pathway.

We further verified the possible regulatory relationship between Rap1b and Nrf2 and the impact of hypoxia on the nuclear translocation of Nrf2 in BGC823 cells under normoxia. Nrf2 was found to be located in the cytoplasm under normoxia (Figure 7A) and in the nucleus under hypoxia (Figure 7B). VEGF-A enhanced nuclear translocation of Nrf2 (Figure 7C). In the negative control group under hypoxia conditions, Nrf2 was located in the nucleus (Figure 8A); however, in the siRap1b (Figure 8B) and siVEGFR2 (Tables 6, 7 and Figures 9A, 8C) groups, there was no Nrf2 entering the nucleus. Under normoxic conditions, silencing Rap1b and VEGFR2, VEGF-A was used to stimulate BGC823 but Nrf2 did not shift to the nucleus (Figure 8D, 8E). This suggested that Rap1b and VEGFR2 could participate in Nrf2’s nuclear translocation. In addition, after siRap1b and siVEGFR2 were silenced, the expression of P-Erk1/2 (Thr202/Thr204) and P-Akt (Thr308) was decreased under hypoxia and VEGF-A induction conditions (Figure 9C). Erk1/2 inhibitor (PD98059) and Akt inhibitor (LY294002) suppressed the nuclear translocation of Nrf2 by VEGF-A (Figure 10). Furthermore, hypoxia promoted the expression of VEGF-A in GC cells (Table 6 and Figure 9B). These results revealed that the hypoxia/VEGF-A-Rap1b/VEGFR2 pathway aided in Nrf2 nuclear translocation and that Erk and Akt signaling cascades were also implicated in Nrf2 nuclear translocation (Figure 11).

## DISCUSSION

Nrf2 is a member of the NF-E2 family and a transcription factor with a basic leucine zipper. Nrf2 participates in the onset and progress of numerous tumors [7]. Herein, Nrf2 was mainly expressed in the nucleus in the GC tissue and was remarkably related with similar clinicopathological characteristics. This reveals that the high expression of Nrf2 promotes the progression of GC; the overexpression of Nrf2 was an independent predictive factor for the survival of GC patients. Similar findings were reported by Kawasaki [13]. However, other reports showed that Nrf2 expression was not associated with pathological parameters (epidemiology, depth of invasion and TNM staging) [14]. The knockdown of Nrf2 expression inhibited the invasion capacity of tumor cell under hypoxic conditions and suppressed the expression of Nrf2, heme oxygenase-1 (HO-1) and HIF-1α. In liver cancer, glioblastoma, esophageal squamous cell carcinoma and breast cancer, Nrf2 promoted tumor cell migration, invasion and distant metastasis [15–18]. These studies suggested that Nrf2 was related to the aggressive behavior of the GC.

HO-1 was predominantly expressed in the cytoplasm, and exhibited similar results to Nrf2 with regards to clinico-pathological characteristics. HO-1 was positively correlated with Nrf2’s expression and can be used as a predictive factor for GC. High expression of Nrf2 along with HO-1 were also related to worse OS outcomes, with the capacity of serving as predictive signatures for prognosis of clear cell renal cell carcinoma patients [19]. Osteopontin-activated Nrf2 signaling enhanced HO-1 expression and migration of glioma cells [20]. Oxysophocarpine (OSC) weakened the expression of Nrf2, as well as HO-1 in oral squamous cell carcinoma (OSCC) cells. In addition, OSC suppressed the growth, migration, infiltration, as well as OSCC cells’ pro-angiogenesis. Furthermore, OSC attenuated the aggressive feature of OSCC cells via Nrf2/HO-1 signaling cascade inactivation [21]. A previous study revealed that ethanol mediated both the activation of Nrf2 and HO-1 to maintain colon cancer cell survival through matrix metallopeptidase 2 (MMP2), MMP9 and VEGF, thus leading to a more aggressive phenotype [22]. Low GRIM-19 (gene linked to retinoid-IFN- triggered mortality 19) is related to dismal survival outcome of individuals with GC. The deficiency of GRIM-19 enhances the metastasis of GC via the oncogenic ROS-Nrf2-HO-1 cascade through a positive-feedback Nrf2-HO-1 loop [23]. Herein, hypoxia up-regulated Nrf2 along with HO-1 expression in GC cells, whilst silencing of Nrf2 expression via siRNA repressed the invasion capacity of tumor cell even in hypoxic culture conditions and repressed Nrf2 expression along with HO-1, *in vitro*. Therefore, Nrf2/HO-1 signal cascade facilitates the GC invasion and metastasis.

This study found that the HIF-1α expression was positively linked to Nrf2expression. Under hypoxic conditions, silencing Nrf2 expression inhibited HIF-1α expression. Our previous study found that in esophageal squamous cell carcinoma cells, silencing Nrf2 expression could inhibit HIF-1α expression and reduce cell invasion under hypoxic conditions [18]. In breast cancer cells, it was shown that the overexpression of Nrf2 augments the expression of Notch1 via G6PD/HIF-1α pathway [24]. The lung cancer mouse model in the chronic intermittent hypoxia (CIH) group enhanced the ß-catenin and Nrf2 nuclear translocation and activated subsequent effectors of Wnt/ß-catenin signaling. CIH also did not trigger the expression of HIF-1α but induced the expression of VEGF. CIH facilitated proliferative along with migratory features of tumors in a mouse model of lung cancer [25]. Taken together, these findings suggest that HIF-1α can be regulated by Nrf2 through different mechanisms under hypoxic and intermittent hypoxic conditions. Nrf2 may affect the TME, making tumor cells more susceptible to invasion and metastasis. Therefore, future studies should highlight the dual functions of the Nrf2-Keap1 cascade in cancer enhancement and repression and describe the mechanisms of its activation, as well as possible treatment approaches based on the context-specific modulation of Nrf2 [7]. In these processes, Nrf2 facilitates the expression of invasion- and migration-related proteins, which reveals that Nrf2 functions as a molecular switch.

Hypoxia is a frequent microenvironment of solid tumors and is considered a powerful driving force, which affects every stage in the process of distant metastasis. Hypoxia and HIF-1α forward tumor migration along with infiltration and distant metastasis via different signaling cascades. The present premise found that under hypoxic conditions, silencing Rap1b or Nrf2 inhibited the invasion of GC cells and co-silencing remarkably inhibited cell invasion, and GC patients in the Nrf2 (+) Rap1b (+) group had the worst prognosis. This suggests that the two may have a synergistic effect. In addition, hypoxic conditions enhanced Nrf2’s nuclear translocation and knockdown of Rap1b inhibited the nuclear translocation of Nrf2. Vascular endothelial growth factor-A (VEGF-A) also accelerated the nuclear translocation of Nrf2 similar to hypoxic. Therefore, hypoxia or VEGF-A may facilitate the nuclear translocation of Nrf2 through Rap1b. In the astrocyte injury model, 2,7,2’-trihydroxy-4,4’7’-trimethoxy-1,1’-biphenanthrene (TTB) potentially activated the Nrf2/HO-1 cascade and protected against oxidative stress in oxygen-glucose deprivation/reoxygenation (OGD/R)-induced astrocytes by stimulating the nuclear translocation, as well as up-regulation of Nrf2 coupled with increased HO-1expression. Treatment with TTB weakened the aggregation of HIF-1α and VEGF expressions triggered by OGD/R. TTB inhibited HIF-1α along with VEGF through the Nrf2/HO-1 signaling cascade and restrained OGD/R-triggered astrocyte oxidative stress [26]. CIH promoted ß-catenin and Nrf2 nuclear translocation and activated subsequent effectors of Wnt/ß-catenin signaling. Besides, CIH did not trigger the expression of HIF-1α but induced the expression of VEGF [25].

Several reports have confirmed that Rap1 regulates the activation of B-Raf/Erk/MAPK [27]. Rottlerin up-regulated Rap1 and the subsequent PI3K/Akt cascade independent of the MAPK/Erk cascade in a dexamethasone-triggered primary open-angle glaucoma cell model [28]. miR-518b promoted trophoblast cell growth via the Rap1b-Ras-MAPK cascade [29]. Adenosine produced by CD73 docked to adenosine A2A receptor-activated Rap1 and promoted Akt phosphorylation in hepatocellular carcinoma cells [30]. Our results showed that knockdown of Rap1b, hypoxia or VEGF-A inhibited the phosphorylation of P-Erk1/2 (Thr202/Thr204) and P-Akt (Thr308). Erk1/2 inhibitor (PD98059) and Akt inhibitor (LY294002) suppressed the nuclear translocation of Nrf2 by VEGF-A. In leukemia cells, combined treatment of AZD0364 (ERK1/2 inhibitor) and ZSTK474 (PI3K inhibitor) reduced nuclear factor-κB and antioxidant protein levels (Nrf2, HO-1, thioredoxin reductase and the reduced glutathione/oxidized glutathione ratio) [31]. Antitumor activity of trastuzumab was enhanced when combined with brusatol in HER2-positive breast cancer cells, which was attributed to the inhibition of the Nrf2/HO-1 and HER2-Akt/Erk1/2 signaling cascades [32]. Cytosolic branched-chain aminotransferase enhances the growth, migration along with the infiltration of triple-negative breast cancer cells through the IGF-1/insulin PI3K/Akt cascade, culminating in the up-regulation of FOXO3a and Nrf2, indicating a novel therapeutic target for breast cancer treatment [33]. These data suggest that the hypoxic/VEGF-A-Rap1b/VEGFR2 cascade facilitates nuclear translocation of Nrf2, Erk, and Akt signal pathways participate in Nrf2’s nuclear translocation.

The activity of VEGFR2 promoted tumorigenicity and indicated poor survival in GC [34]. VEGF/VEGFR2 cascade is the central treatment target of the anti-angiogenic treatment for numerous cancers. Hypoxia enhanced NRf2’s nuclear translocation and the knockdown of VEGFR2 inhibited the nuclear translocation of Nrf2. VEGF-A also enhanced Nrf2’s nuclear translocation similar to hypoxia. Besides, knockdown of VEGFR2, hypoxia or VEGF-A inhibited the phosphorylation of P-Erk1/2 and P-Akt (Thr308). Hypoxia or VEGF-A facilitates the nuclear translocation of Nrf2 through VEGFR2. Apatinib, an inhibitor of targeting VEGFR2, has been approved for third-line treatment of advanced GC [35]. Ramucirumab, the human immunoglobulin G1 monoclonal antibody receptor antagonist of VEGFR2, has been approved for the treatment of gastric/gastroesophageal junction and non-small-cell lung and metastatic colorectal cancers [36]. However, these two drugs are used as a posterior-line treatment of GC; there are still numerous challenges in the treatment of GC via inhibiting VEGFR2.

In conclusion, our findings highlight the expression profile of Rap1b and Nrf2 in malignant behavior and poor prognosis. Hypoxic conditions promoted the aggressive behavior of GC cells through the up-regulation of Rap1b and Nrf2. Hypoxic/VEGF-A-Rap1b/VEGFR2 pathway facilitated the nuclear translocation of Nrf2. Targeting Rap1b and Nrf2 expression may be promising prognostic biomarkers, as well as novel therapeutic strategies in GC.

## MATERIALS AND METHODS

### Materials

Diaminobenzidine (DAB) was provided by Golden Bridge International (Beijing, China) and DMEM was provided by Life Technologies (Grand Island, NY, USA). FBS, Lipofectamine 2000 along with TRIzol reagent were acquired from Invitrogen (Carlsbad, CA, USA). The Superscript II reverse transcriptase kit was supplied by Takara Bio Inc. (Otsu, Japan), as well as the ECL reagent along with PVDF membranes were commercially acquired from Merck Millipore (Darmstadt, Germany). The RIPA lysis buffer kit was acquired from Santa Cruz Biotechnology (Santa Cruz, CA, USA) and the protein assay kit was obtained from Bio-Rad (Hercules, CA, USA). The membrane infiltration culture system was purchased from Corning (Corning, NY, USA). CoCl2 coupled with other reagents were supplied by Sigma (St. Louis, MO, USA).

### Tissue specimens

Tissue specimens were taken from consecutive surgical cases of 178 GC patients in the Department of Surgical Oncology, the First Affiliated Hospital, School of Medical, Xi’an Jiaotong University and the Department of Surgical Oncology, the 215th Hospital of Shaanxi province, between 2004 and 2009. There are 125 male and 53 female patients, aged between 25 and 81 years old. The clinicopathological data from these patients are shown in Table 2. There were 77 patients (43.25%) whose tumors penetrated series or involved adjacent structures, 129 subjects (72.47%) exhibited lymph node metastases and 40 subjects (22.47%) harbored distant metastasis. 26 subjects were treated with total gastrectomy, whilst the remaining 152 subjects were treated with partial resection of tumor lesions. None of the 178 subjects received any neoadjuvant prior to surgery. However, there were 108 of 178 subjects receiving fluorouracil-based postoperative adjuvant chemotherapy. This study was approved by the Ethic Committee of the First Affiliated Hospital, School of Medicine, Xi’an Jiaotong University.

### Immunohistochemistry

The tissue sections were deparaffinized and rehydrated, before antigen retrieval in a citrate buffer (pH 6.0) in the microwave. After that, we probed the sections with a goat polyclonal antibody against Nrf2 (1:600 Santa Cruz, CA, USA) or HO-1 (1:600 Biosynthesis Biotechnology, Beijing, China) overnight at 4° C. The secondary antibodies (SP-9001 along with PV-9003; Golden Bridge Intl., Beijing, China) were utilized to enlarge the signal. Visualization of the sections was done with 0.02% DAB solution to generate the positive color reaction, and counterstained with hematoxylin.

### Cell lines and culture

MKN28, SGC7901 along with BGC823 human cell lines were supplied by Cell Bank of Shanghai (Shanghai, China) and maintained in DMEM containing high glucose and enriched with 10% FBS inactivated with heat. The cells at the logarithmic phase were used in the experiments.

### Transwell invasion assay

Briefly, re-suspension of GC cell transfects of the control or sinNrf2 (3-5 × 104/well) was done in of serum-free DMEM (200 μL) enriched with CoCl2 (150 or 200 μM). The resuspension was subsequently introduced to the upper transwell chambers. In addition, we introduced DMEM (600 μL) enriched with FBS (20% (v/v) along with CoCl2 (150 or 200 μM) to the lower transwell compartments to serve as a chemical attractant. The assays were run under 37° C and 5% CO2 for 24 hours. Afterwards, a cotton swap was utilized to remove the top cells, and fixation of the tumor cells which infiltrated the lower surface done with 4% PFA, followed by staining (in 1% crystal purple). For each insert, we selected 5 random fields for visualization with a light microscope. Each experiment was implemented in triplicate and repeated once.

### siRNA and transfection

GenePharma Co. (Shanghai, China) devised and synthesized the small interference RNA molecules targeting human Nrf2 and VEGFR2. The following were the targeted sequences:

The GC cells were cultured and inserted with the negative control or Nrf2 siRNAs (VEGFR2 siRNAs) via a Lipofectamine 2000 in the light of the instructions of the manufacturer.

### RNA isolation and qRT-PCR

Purification of total cellular RNA from GC cells was done with the TRIzol reagent. After that, cDNA was generated from the RNA with the Superscript II Reverse Transcriptase kit as described by the manufacturer. qPCR was done using the primers:

The amplification conditions were 95° C for 30 s and 30 cycles of 95° C for 5 s, 60° C for 30 s and 72° C for 60 s for 30 cycles. The quantity of each transcript was standardized to that of GAPDH.

### Protein extraction and western blot

Lysis of cells was done in cold RIPA lysis buffer, and proteins quantitated with the Bio-Rad protein assay. Equivalent amounts of protein samples were loaded and separated by SDS/PAGE and transfer-embedded onto a PVDF membrane. The blots were inoculated with antibodies against Nrf2, HO-1 and HIF-1α, Erk (1:600), P-Erk (Thr202/Thr204, 1:600), Akt (1:1000) and P-Akt (Thr308, 1:1000). Afterwards, blocking of the membranes was done in TBS with tween-20 enriched with 5% nonfat dry milk. Thereafter, the membranes were inoculated overnight with HRP-labelled antibodies at RT (room temperature). Detection of blots was done with the ECL detection system and analyzed densitometrically via the Quantity One software (Bio-Rad, Hercules, CA, USA).

### Immunofluorescence staining

To explore Nrf2’s nuclear translocation, fluorescence immunocytochemistry was conducted. GC cells were planted on glass coverslips and were divided into several groups. Fixation of cells (in 4% PFA) was done for 15 minutes at RT and rinsed with cold PBS three times, followed by permeabilization for 10 minutes (in 0.5% Triton X-100). To investigate the cellular localization of Nrf2, cells were probed overnight with the primary antibodies against Nrf2 at 4° C. After rinsing with PBS, cells were inoculated for one hour in Cy3-labelled goat anti-rabbit IgG (secondary antibody) at RT. Nuclei staining was done in 1 μg/ml of DAPI for 5 min in the dark, and then analyzed by a fluorescence microscope (Olympus IX71).

### Statistical analysis

Statistical analyses were implemented by the SPSS v20.0 (Chicago, IL, USA). The chi-squared test was used to determine the association between Nrf2 and HO-1 expression levels and clinicopathological variables. The Spearman's rank-order was adopted to assess the relationship of HO-1 expressions with Nrf2 expression in tissue specimens. Survival plots were performed via the Kaplan-Meier approach, which was then compared with the log-rank test. We adopted the Co’s proportional hazard approach to conduct multivariate analyses for the evaluation of predictive indicators. Data from separate replicate trials were pooled for each treatment, as well as for the control, and the findings were presented as means ± standard error. For normally distributed variables, the Student’s t-test or one-way analysis of variance was used. A p-value of less than. 05 signified statistical significance.
